# Supplementary material for: Regulation of axonal morphogenesis by the mitochondrial protein Efhd1
Source: Life Sci Alliance. 2020 May 15;3(7):e202000753. doi: 10.26508/lsa.202000753 (PMC7232985; doi:10.26508/lsa.202000753)

Figure S1

Lkb1 WT and KO soma and axons: ACC and AMPK phoshorilation level

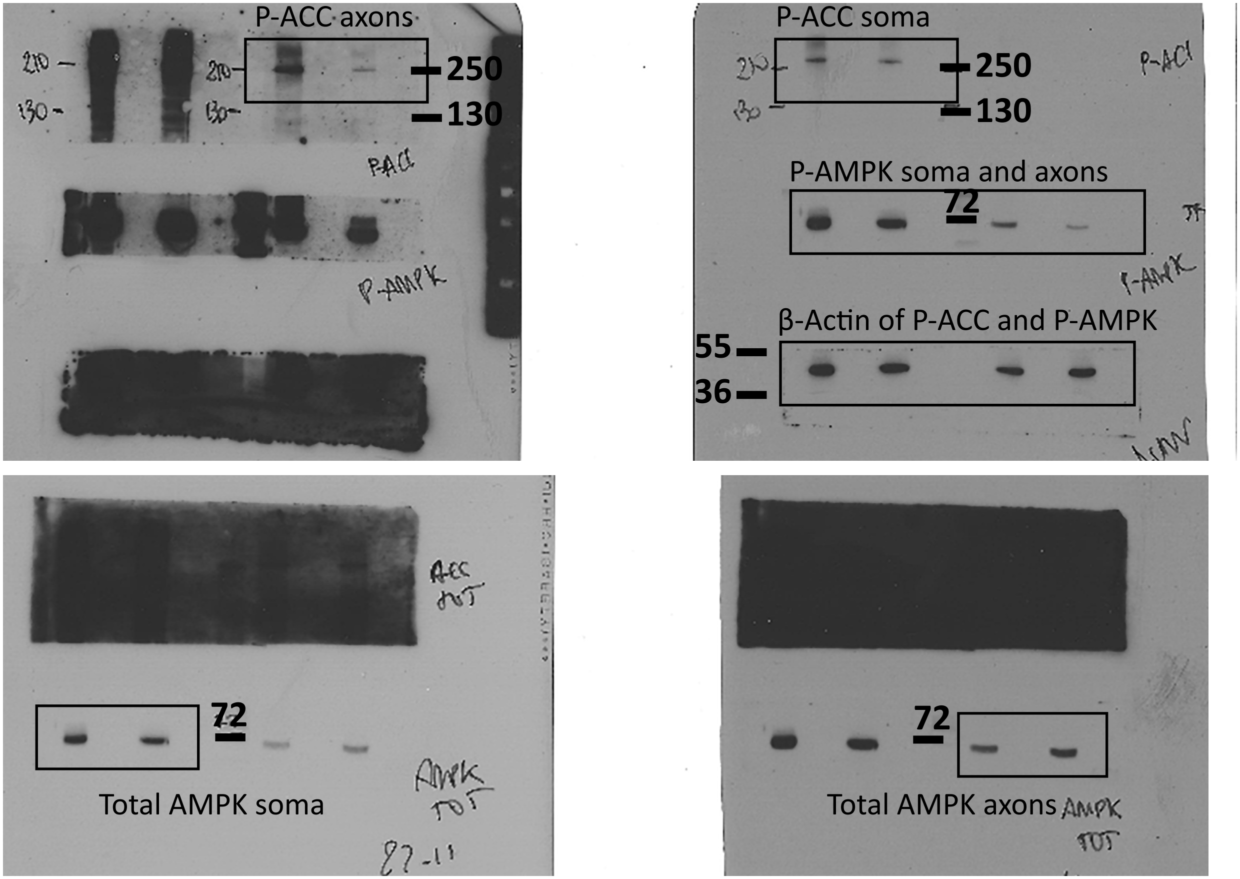

Total ACC soma and axons

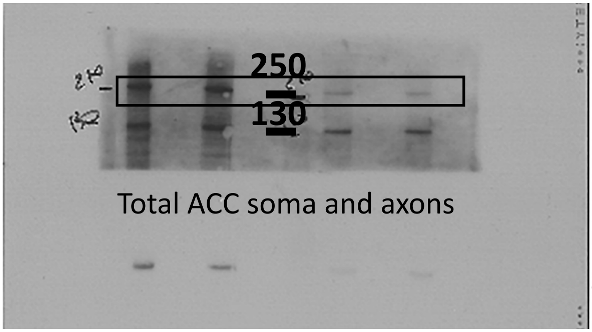

Lkb1 WT and KO DRGs: Lkb1 protein level

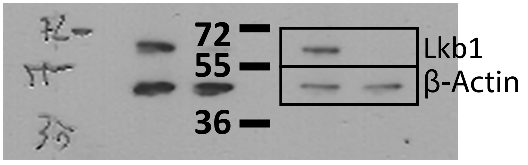

Supplement: Supplementary file 1 [file LSA-2020-00753_SdataFS1.pdf]
