# Supplementary material for: Regulation of axonal morphogenesis by the mitochondrial protein Efhd1
Source: Life Sci Alliance. 2020 May 15;3(7):e202000753. doi: 10.26508/lsa.202000753 (PMC7232985; doi:10.26508/lsa.202000753)
Supplement: Supplementary file 2 [file LSA-2020-00753_SdataF1.pdf]

# Figure 1

Lkb1 WT and KO soma and axons: Efhd1 protein level

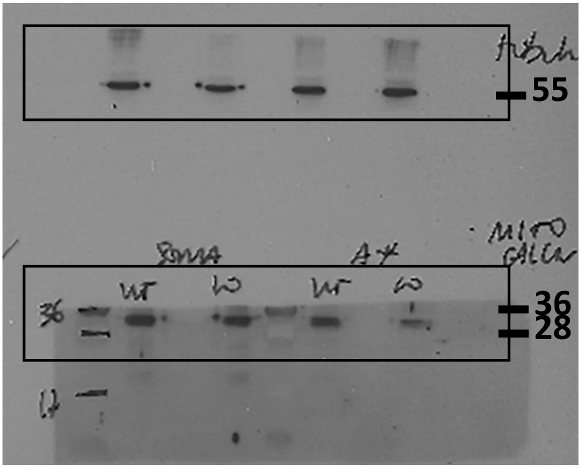


α-βIII-tubulin of Efhd1

Efhd1 protein level in soma and axons

WT soma and axons +/- 8h Compound C treatment:
Efhd1 protein level

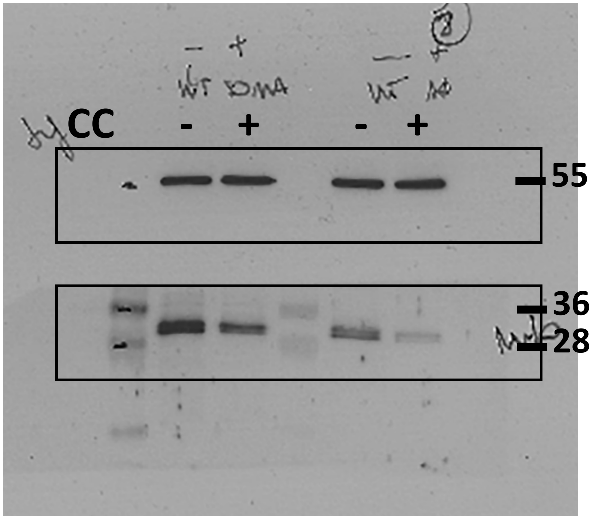


α-βIII-tubulin of Efhd1

Efhd1 protein level in soma and axons
