# Supplementary material for: Regulation of axonal morphogenesis by the mitochondrial protein Efhd1
Source: Life Sci Alliance. 2020 May 15;3(7):e202000753. doi: 10.26508/lsa.202000753 (PMC7232985; doi:10.26508/lsa.202000753)

Figure S2

Lkb1 WT and KO soma and axons : TOM20 protein level

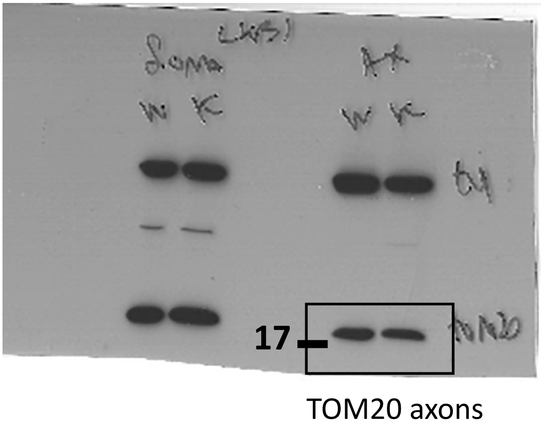

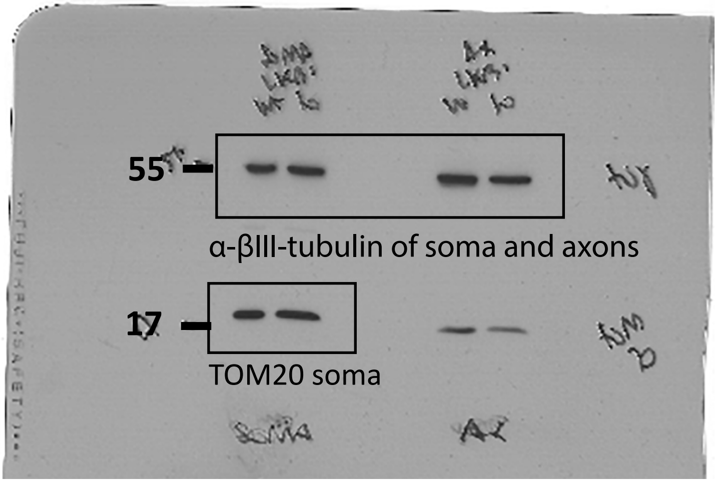

WT soma and axons +/- 8h Compound C : TOM20 protein level

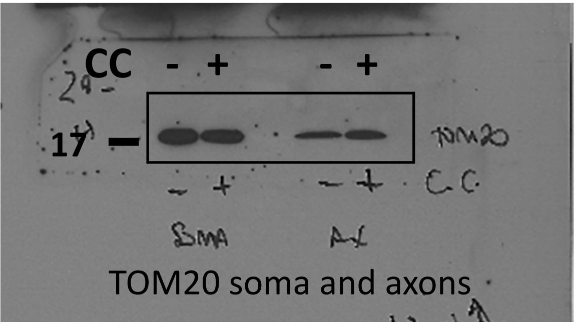

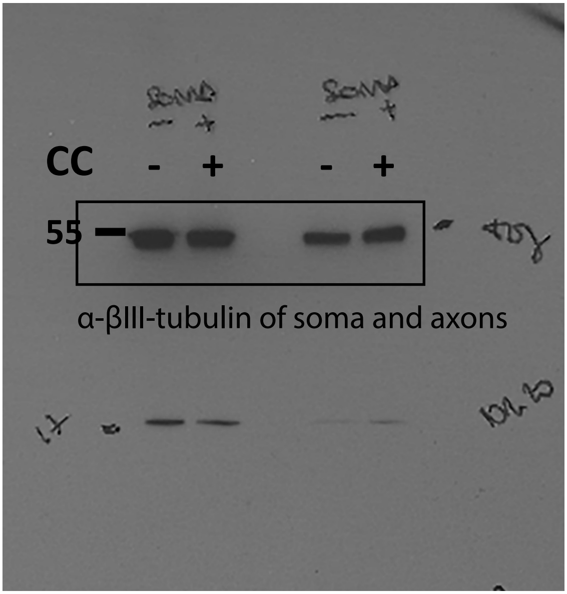

Supplement: Supplementary file 4 [file LSA-2020-00753_SdataFS2.pdf]
