# Supplementary figures and images for: Regulation of axonal morphogenesis by the mitochondrial protein Efhd1
Source: Life Sci Alliance. 2020 May 15;3(7):e202000753. doi: 10.26508/lsa.202000753 (PMC7232985; doi:10.26508/lsa.202000753)

Figure S3  
Efhd1 WT and KO DRGs: Efhd1 protein level

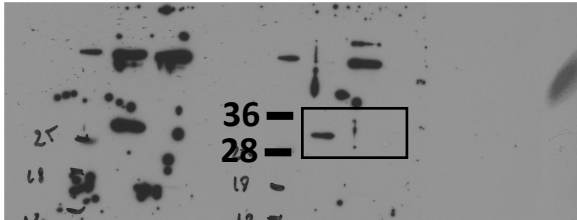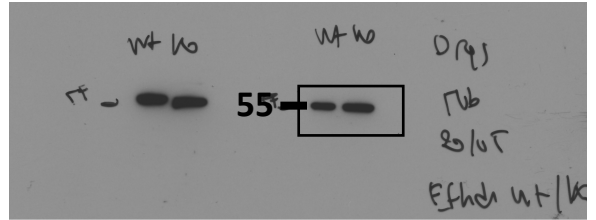

Supplement: Supplementary file 5 [file LSA-2020-00753_SdataFS3.pdf]
