# Supplementary material for: Regulation of axonal morphogenesis by the mitochondrial protein Efhd1
Source: Life Sci Alliance. 2020 May 15;3(7):e202000753. doi: 10.26508/lsa.202000753 (PMC7232985; doi:10.26508/lsa.202000753)

Figure 5, Figure S5

Efhd1 WT and KO soma and axons E13.5

P-AMPK soma

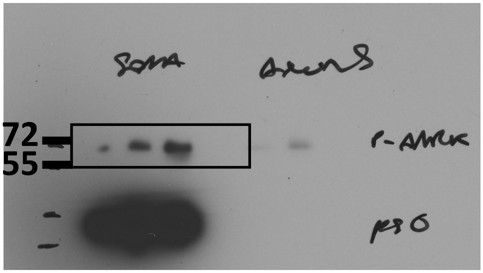

AMPK total soma+ axons

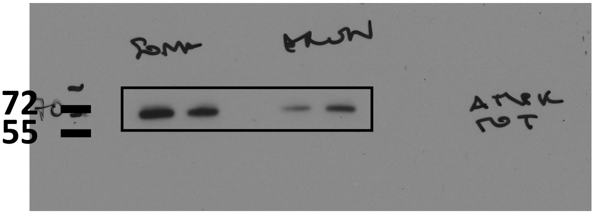

P-AMPK axons

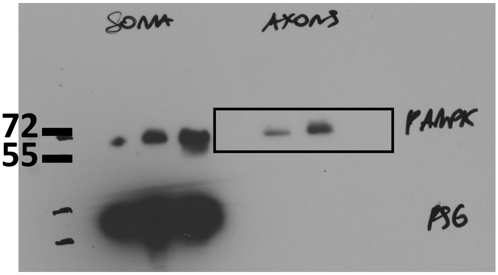

α-βIII-tubulin soma+ axons

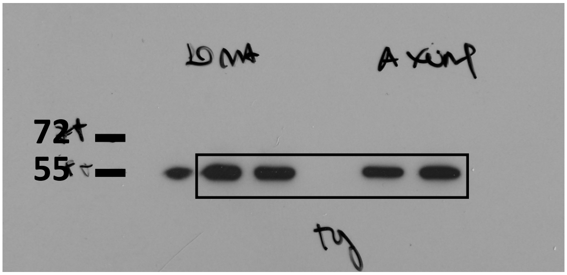

P-ACC soma + ACC total axons

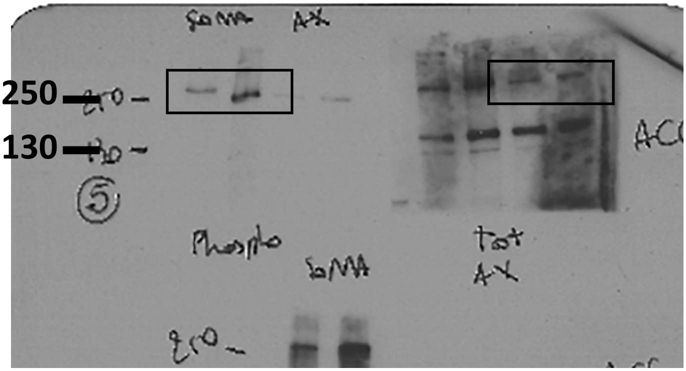

ACC total soma

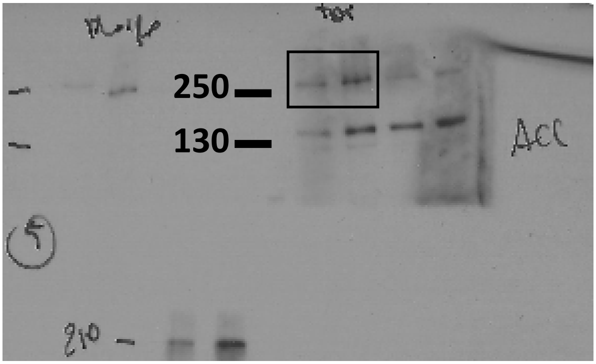

P-ACC axons

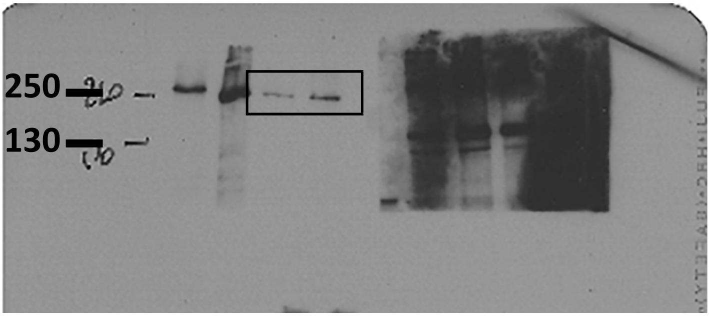

α-βIII-tubulin soma+ axons

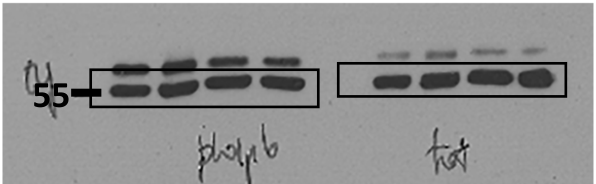

α-βIII-tubulin of P-ACC

α-βIII-tubulin of total-ACC

P-Ulk soma

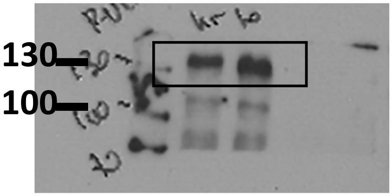

P-Ulk axons

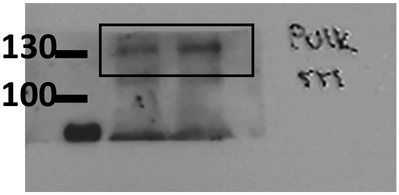

Ulk total soma

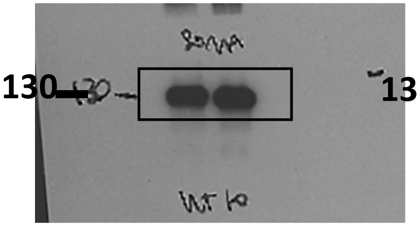

Ulk total axons

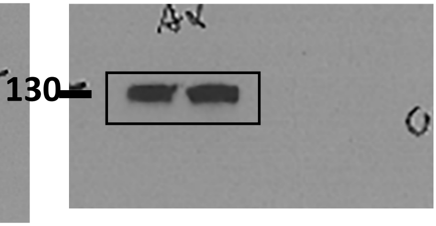

α-βIII-tubulin soma

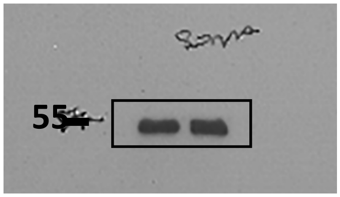

α-βIII-tubulin axons

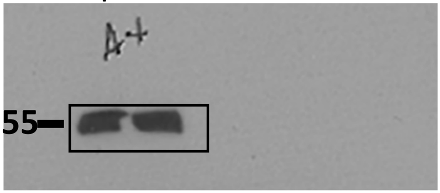

Efhd1 WT and KO DRGs E 17.5

P-AMPK E17.5

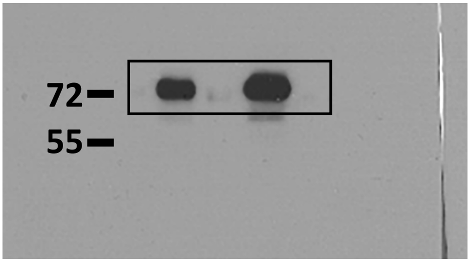

AMPK total E17.5

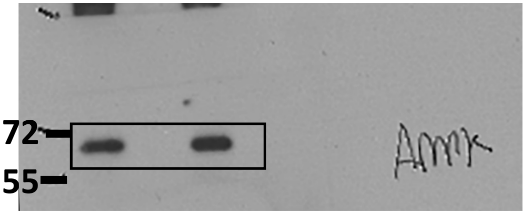

α-βIII-tubulin of AMPK

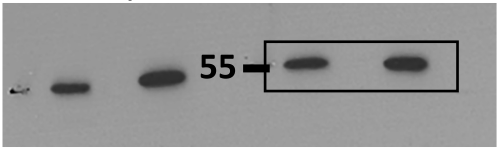

P-Ulk E17.5

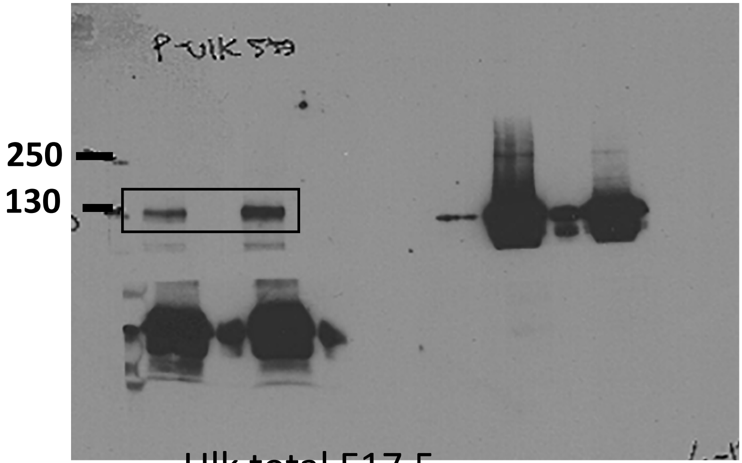

Ulk total E17.5

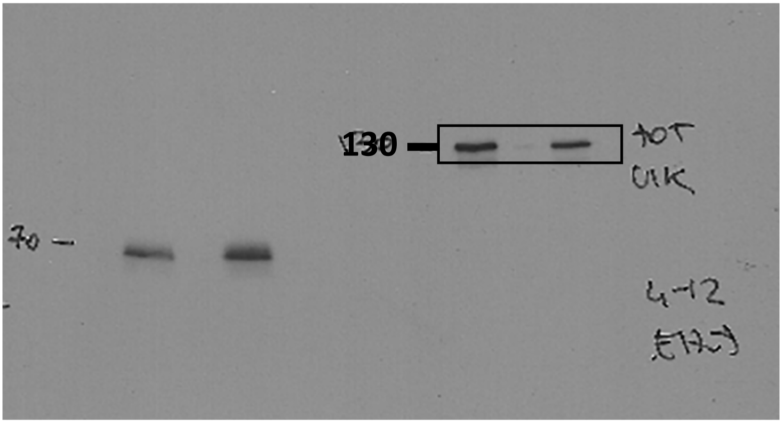

α-βIII-tubulin P-Ulk e17.5

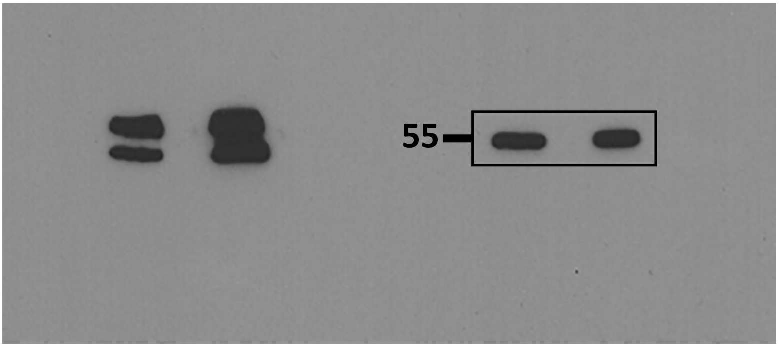

Efhd1 WT and KO DRGs E 13.5

LC3I-II -/+ Bafilomycin

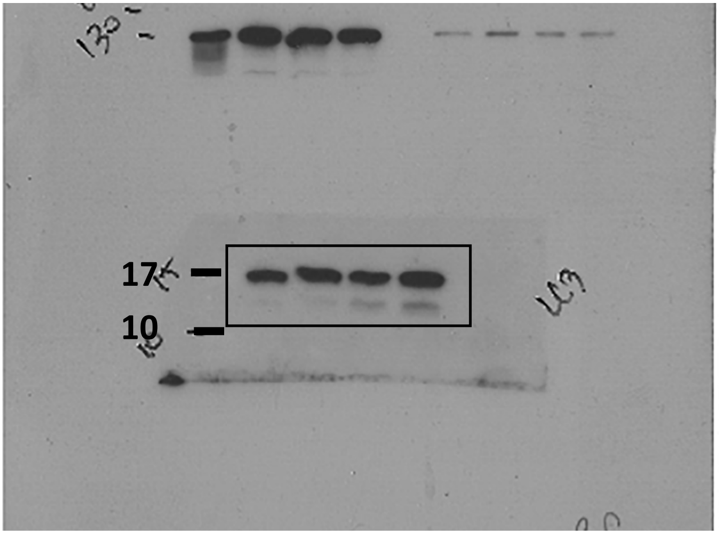

α-βIII-tubulin of LC3I-II

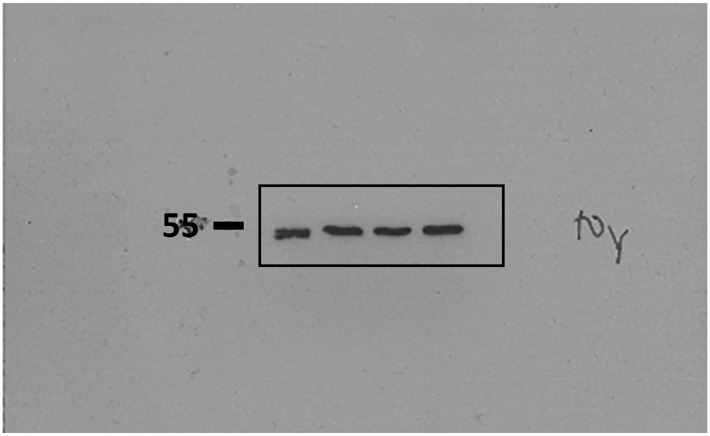

Supplement: Supplementary file 6 [file LSA-2020-00753_SdataF5.pdf]
